# Continuously applying compost for three years alleviated soil acidity and heavy metal bioavailability in a soil-asparagus lettuce system

**DOI:** 10.3389/fpls.2022.972789

**Published:** 2022-08-03

**Authors:** De Chen, Xuezhu Ye, Yugen Jiang, Wendan Xiao, Qi Zhang, Shouping Zhao, Sainan Shao, Na Gao, Miaojie Huang, Jing Hu

**Affiliations:** ^1^State Key Laboratory for Managing Biotic and Chemical Threats to the Quality and Safety of Agro-products, Key Laboratory of Information Traceability for Agricultural Products, Ministry of Agriculture and Rural Affairs of China, Zhejiang Academy of Agricultural Sciences, Institute of Agro-product Safety and Nutrition, Hangzhou, Zhejiang, China; ^2^Agricultural Technology Extension Center of Fuyang District, Hangzhou, Zhejiang, China

**Keywords:** bioavailability, heavy metal accumulation, heavy metal migration, organic fertilizer, soil acidity amelioration, uptake and translocation of heavy metals

## Abstract

Soil acidification and heavy metal pollution are two common barrier factors threatening plant growth and agro-product quality. Applying manure compost is promising to alleviate soil acidity, while it may increase heavy metal accumulation in soil. In a 3-year field experiment, compost was applied for 12 consecutive harvest seasons at 15, 30, and 45 t ha^−1^ in a slightly acidic soil. Samples were taken at the twelfth season to examine the changes of soil properties, vegetable productivity, heavy metal accumulation and bioavailability in the soil-asparagus lettuce system. The results showed that the pH values of the topsoil were increased by 0.49–0.75 units in compost added soils compared with no compost control, soil organic matter (SOM) contents and cation exchange capacity (CEC) were increased by 34–101% and 43–44%, respectively. The soil nutrient contents were also increased in compost treatments. Continuously applying compost increased Cd, Cu, and Zn concentrations in topsoil by up to 32, 20, and 22% and decreased Pb by 10%, while soil available Cd and Zn concentrations were reduced by up to 54 and 86%, and available Cu was increased by 19–63%. The biomass of asparagus lettuce was increased by 30–59% in compost treatments, with Cd and Zn concentrations in the plant tissues reduced by 28–50% and 14–67%. Cu concentrations in the lettuce shoots were increased by 20–39%. The concentration factor and total uptake of Cd and Zn in lettuce were effectively reduced in compost treatments. Cd was more prone to be taken up, translocated and accumulated from soil to the lettuce plant than the other heavy metals. Continuously applying compost over 3 years increased soil pH, SOM, CEC, nutrient contents, and lettuce productivity, decreased Cd and Zn bioavailability in the soil-lettuce system, while posing a risk of increasing heavy metal accumulation in topsoil.

## Introduction

Soil degradation has been a worldwide concern which poses great threat to the sustainability of global agriculture and food security (Lal, 2001; Núñez-Delgado et al., 2020). Soil acidification in China is a severe problem caused by acid precipitation, overuse of chemical fertilizers and unreasonable cultivation (Guo et al., 2010), causing deterioration of soil physical and chemical properties, reduction of crop yield, as well as increasing heavy metal bioavailability in soil–plant system (Matsuyama et al., 2005; Kicińska et al., 2022). In the last several decades, the use of chemical fertilizers has dramatically increased in China for high yield (Zhang et al., 2012), which caused environmental problems such as soil acidification, greenhouse gases mitigation, and decrease of soil and agro-product quality (Liu and Zhang, 2011; Zeng et al., 2017). The status of soil heavy metal pollution in Southern China was a matter of concern, while soil acidification can further increase the bioavailability of heavy metals (Zhao et al., 2015). Therefore, measures should be taken to deal with soil acidification caused by excessive use of chemical fertilizer. Application of organic or inorganic amendments into soil is an efficient measurement to cope with soil acidification, increase soil fertility, and reduce plant uptake of heavy metals (Lu et al., 2020).

With the rapid development of livestock and poultry breeding industry, large quantities of manure was produced every year in China, which poses a threat to the environment (Tilman et al., 2002; Thornton, 2010). Composting of animal manure is a promising measurement not only providing an solution for tackling the large quantities of produced manure but also effective in improving soil fertility as an organic fertilizer (Iqbal et al., 2019; Martins et al., 2022; Rizzo et al., 2022). In China, there is a long history where farmers applied manure or compost to soil as fertilizer to improve soil quality and crop productivity, contributing to the sustainable development of agriculture throughout Chinese history (Ellis and Wang, 1997). Currently, the substitution of chemical fertilizers with organic fertilizers is recommended as one of the measures to deal with the overuse of chemical fertilizers in China (Xin et al., 2017; Wang et al., 2018). Manure compost is rich in organic matter, nitrogen, phosphorus, potassium, and other minerals (Sullivan et al., 1997; Chastain et al., 1999), which are beneficial for improving soil physicochemical properties, soil fertility (Lehmann et al., 2003), and crop productivity (Steiner et al., 2007). The application of organic fertilizer is beneficial to alleviating soil acidification compared to chemical fertilizers (Dai et al., 2021). In addition, increased soil pH, Soil organic matter content (SOM), and cation exchange capacity (CEC) assist in stabilizing soil heavy metals (Kumar et al., 2013), while the accumulation of heavy metals in soil is a matter of concern as a result of continuous application of manure compost (Xin et al., 2017).

Although many studies have reported the effect of manure or compost on heavy metal accumulation and corresponding plant uptake, the results have been inconsistent (Zhao et al., 2014; Wang et al., 2020). For instance, Wang et al. (2020) reported that after 16 years of pig manure application, Zn and Cd concentrations in the soil and peanut kernels were significantly higher compared with chemical fertilizer treatment. Hussain et al. (2021) reported that the Cd, Zn and Cu concentrations in soil and rice grains were significantly increased after application of swine manure for 27 years compared with chemical fertilizer treatment, and the EDTA-extractable Cd, Zn, and Cu in soil were also increased. Wan et al. (2020) reported that the application of chicken or swine manure after four years significantly reduced the Cd and Pb contents in rice grain by 7.8–79.3% and 7.2–59.4%, the exchangeable Cd and Pb fractions, and the DTPA-extractable Cd and Pb in the soil were also decreased. The inconsistent effects of manure application on heavy metal bioavailability are probably due to the differences of heavy metal contents in the manure, the application rate, frequency and the duration of the experiment, as well as the differences of soil properties and plant types. In addition, a limited number of studies examined the accumulation and migration of heavy metals at different depths of the soil profile (Zhou et al., 2020). Therefore, it is still necessary to comprehensively evaluate the long-term impacts of specific types of manure or compost on alleviating soil acidity, improving soil fertility, and regulating the accumulation and bioavailability of heavy metals in specific situation.

Asparagus lettuce is a popular vegetable in China. However, there are few studies systematically estimating the uptake and translocation of heavy metals by asparagus lettuce, especially in soils treated by long-term application of organic fertilizer. In this study, a field experiment was conducted through continuously applying manure compost in a field of vegetables over a period of 3 years. The purpose was to investigate the effect of the compost application at various intensities on soil acidity and fertility regulation, heavy metal accumulation and availability in different soil layers, as well as the biomass and heavy metal uptake and translocation by asparagus lettuce, in order to analyze the potential balance of mitigating soil acidification, the improvement of soil fertility, and the regulation of heavy metal accumulation and bioavailability.

## Materials and methods

### Location and manure

A 3-year field experiment was conducted in the Fuyang District, Hangzhou, Zhejiang Province, China. The climate is subtropical monsoon, with a mean annual temperature of 16.4°C and an average annual precipitation of 1,814 mm. The area studied was on a farm where local farmers have grown vegetables for many years. The soil type is percolated paddy soil. The compost used in this study was purchased from Zhejiang Ruijue Biotechnology Co., Ltd., Zhejiang Province, China. The main components of the compost were pig manure and silkworm excrement, which were the common solid waste of breeding industry in Zhejiang Province. The agronomic characteristics of the compost and the paddy soil are presented in Table 1.

**Table 1:** Basic properties of the tested soil and compost.

Characteristics	Soil	Compost
pH	6.40	8.2
Clay	22.1	–
Sand	31.7	–
Silt	46.2	–
Texture	Loam	–
Bulk density (g cm^−3^)	1.21	–
CEC (cmol kg^−1^)	16.7	–
Organic matter (g kg^−1^)	32.0	345
Total N (g kg^−1^)	2.40	15.2
Total P (P_2_O_5_, g kg^−1^)	4.10	15.6
Total K (K_2_O, g kg^−1^)	–	14.5
Available P (mg kg^−1^)	1296.7	–
Total Cd (mg kg^−1^)	0.54	0.25
Total Pb (mg kg^−1^)	43.8	15.4
Total Cu (mg kg^−1^)	139.8	80
Total Zn (mg kg^−1^)	429.2	130
Total Cr (mg kg^−1^)	48.0	16.7

### Experimental design

The field experiment was conducted in 2013. Four treatments were included in the present study, only organic fertilizer was applied in each growing season at rates of 0, 15, 30, and 45 t ha^−1^ on a slightly acidic soil, and labeled as CK, T1, T2, and T3, respectively. There were 3–4 growing seasons a year according to the type of vegetables planted. The organic fertilizer were applied 2 weeks before the vegetable seedlings were transplanted, and was then mixed thoroughly by plowing it to a depth of 0–20 cm. Each plot comprised an area of 25 m^2^ (5 m × 5 m). In 2016, asparagus lettuce (*Lactuca sativa* L. var. *angustata Irish ex Bremer*) was planted on June 10 and harvested on October 25, which was the twelfth season of vegetable planting since the experiment was established. The other field managements were consistent with the local practices except for fertilization.

### Sampling and analysis

At harvest time, five whole plant samples were randomly selected from each plot. When delivered to the lab, the plants were washed with deionized water and then separated into leaves, shoots, and roots. Each component was weighed before oven-drying at 105°C for 30 min, then oven-dried further at 80°C. The dried plant tissue samples were ground using a stainless-steel mill, then mixed, homogenized, and stored in air-tight polyethylene bags prior to analysis.

Soil samples were collected for each treatment after harvesting. Composite samples consisting of five undisturbed core samples at three depths (0–20 cm, 20–40 cm, and 40–60 cm) within the soil profile were randomly collected using a stainless-steel core sampler. Each composite sample was sealed in a plastic bag, transported to the laboratory, and air-dried. Plant detritus and other fragments were removed before the soil samples were ground and passed through 2 and 0.15 mm nylon sieves. The former part of soil was used to determine soil pH, available nutrients, and extractable heavy metals, and the latter part was used to analyze SOM, total content of nutrients and heavy metals.

The soil properties were analyzed according to the procedures described by Lu (2000). Soil pH was determined using a precision pH meter (PHS-3C, Rex, Shanghai, China) with a soil:water ratio of 1:2.5 (m:v). Soil organic matter content (SOM) was measured using a modified Walkley-Black titrimetric procedure after potassium dichromate digestion. Soil CEC was determined by the ammonium acetate method, and soil total nitrogen content (TN) was measured using the semi-micro Kjeldahl method, while the alkali-hydrolyzed nitrogen (AN) was tested by the alkaline hydrolysis diffusion method. The total phosphorus content (TP) of the soil was analyzed colorimetrically after wet digestion with H_2_SO_4_–HClO_4_ mixture_._ To determine the available phosphorus content (AP), the soil sample was extracted using a 0.03 M NH_4_F–0.025 M HCl solution and measured colorimetrically according to the formation of the blue phosphomolybdate complex. The total concentrations of Cd, Pb, Cu, and Zn in the soil were determined after digestion with HNO_3_–HClO_3_–HF (5:1:1, v:v:v) in an automatic digester (Vulcan 84, Questron Technology Corporation, Mississauga, ON, Canada). The heavy metals present in the soil were determined after being extracted with 0.01 M CaCl_2_ (m:v, 1:10). The plant samples were digested with HNO_3_–H_2_O_2_ in a microwave digester (Mars X, CEM Corporation, North Carolina, United States of America), then heavy metal concentrations in the digestion solution were analyzed using ICP-MS (X-series 2, Thermo Fisher Scientific Inc., Massachusetts, United States of America).

Reagent blanks and standard reference materials (SRM) were included in each batch to ensure accuracy and precision. A dark brown soil certified as GBW07401 (GSS-1) was selected as the SRM for soil, with heavy metal recovery rates ranging 88–114%. Rice flour (GBW10045, GSB-23) and celery (GBW10048, GSB-26) were used as SRMs for the plant samples, with the recovery rates of the four heavy metals being between 89 and 105%. All reference materials were purchased from the National Research Center for Standards in China.

The translocation factor of heavy metals from root to shoot (TF_S/R_) and shoot to leaf (TF_L/S_) is defined as the ratio of the concentration of each heavy metal in plant shoots to roots and leaves to shoots, respectively. The bioconcentration factors were defined as the ratios of the concentration of each heavy metal in leaves or shoots to that in soil, denoted as BCF_Leaf/Soil_ and BCF_Shoot/Soil_, respectively. The quantity of heavy metal uptake in asparagus lettuce leaves, shoots, and roots was calculated as the heavy metal concentration multiplied by the corresponding biomass for each component. The total heavy metal uptake by the plants equals the sum of the Cd uptake in the leaves, shoots, and roots of the lettuce.

### Statistical analysis

All data are presented as mean plus or minus one ± standard deviation (mean ± SD, *n* = 3). One-way analysis of variance (ANOVA) was used to explore the differences between various organic fertilizer treatments or within different soil layers. Two-way ANOVA analysis was employed to determine the effects of organic fertilizer and soil layers with different depth on heavy metal accumulation and availability in soil. A statistical analysis was conducted using SPSS (version 20.0; IBM, Armonk, NY, United states of America). If significant differences were found among treatments, individual means were compared using the least significant difference test (LSD) at a level of 0.05. The figures were drawn using Microsoft Excel 2016 and Sigma Plot 12.0 (Systat Software Inc. San Jose, CA, United States of America).

## Results

### Soil properties

Table 2 shows that compost application has positive effects on soil physiochemical properties and nutrient content, especially in the topsoil. The pH values of the topsoil were increased by 0.54, 0.75 and 0.49 units in the T1, T2, and T3 treatments respectively, compared to CK. Soil pH in the 20–40 cm layer was increased by 0.32 and 0.37 units in the T2 and T3 treatments. The SOM content in the topsoil was increased by 34, 79, and 101%, respectively for the T1, T2 and T3 treatments, and the SOM in the subsurface layer (20–40 cm) was increased by 71 and 54%, respectively for the T2 and T3 treatments. Furthermore, the SOM content in the topsoil layer was much higher than that in the subsurface layer and the deep soil (40–60 cm). The soil CEC in the topsoil for the T2 and T3 treatments was 43 and 44% higher compared to CK. The soil TN was increased by 37, 97, and 95%, respectively for T1, T2, and T3, compared with CK, where it only increased in the topsoil. The addition of the compost generally increased the AN in each layer of soil, especially for the T2 and T3 treatments. The TP content in the topsoil were increased by 13–44% with compost treatments compared to CK. It only increased by 37% for T2 in the subsurface layer, and there were no significant changes of TP in the deep soil. There were no significant changes of AP in the topsoil, while there were increases of 9–67% in the subsoil. In the deep soil, the AP content was increased by 45% only in T3, compared to CK. Finally, compost application reduced the bulk density (BD) by 16, 18, and 25%, respectively for the T1, T2, and T3 treatments, in the topsoil.

**Table 2:** Effects of compost application on soil properties (mean ± SD, *n* = 3).

Depth (cm)	Treatment	pH	SOM (g kg^−1^)	CEC (cmol kg^−1^)	TN (g kg^−1^)	AN (mg kg^−1^)	TP (g kg^−1^)	AP (mg kg^−1^)	BD (g cm^−3^)
0–20	CK	6.43 ± 0.07c	32.2 ± 0.60d	16.7 ± 0.1b	2.43 ± 0.17c	99.8 ± 6.2d	4.06 ± 0.13c	1296.7 ± 39.6a	1.21 ± 0.06a
	T1	6.97 ± 0.22ab	43.2 ± 2.13c	19.0 ± 1.6b	3.33 ± 0.57b	147.5 ± 21.2c	4.59 ± 0.45b	1299.1 ± 44.9a	1.02 ± 0.06b
	T2	7.18 ± 0.02a	57.5 ± 1.36b	23.9 ± 2.2a	4.79 ± 0.10a	206.1 ± 15.6b	5.63 ± 0.08a	1362.9 ± 58.1a	0.99 ± 0.05bc
	T3	6.92 ± 0.09b	67.6 ± 2.31a	24.0 ± 1.4a	4.73 ± 0.10a	263.6 ± 30.2a	5.84 ± 0.14a	1318.9 ± 63.3a	0.91 ± 0.03c
20–40	CK	6.23 ± 0.13b	11.2 ± 0.14c	11.6 ± 0.9a	0.93 ± 0.09a	41.7 ± 1.3c	1.39 ± 0.17b	550.3 ± 33.2c	–
	T1	6.40 ± 0.14ab	12.2 ± 1.22bc	12.5 ± 2.1a	1.17 ± 0.38a	52.8 ± 12.7bc	1.54 ± 0.19ab	599.4 ± 88.9c	–
	T2	6.55 ± 0.09a	19.1 ± 5.66a	12.9 ± 2.2a	1.14 ± 0.16a	65.5 ± 7.9b	1.90 ± 0.33a	802.9 ± 17.7b	–
	T3	6.60 ± 0.22a	17.2 ± 0.73ab	12.5 ± 1.2a	1.23 ± 0.05a	81.3 ± 6.1a	1.63 ± 0.23ab	916.9 ± 72.1a	–
40–60	CK	6.4 1 ± 0.26a	7.64 ± 0.31a	11.7 ± 1.7ab	0.76 ± 0.03a	31.5 ± 4.7c	0.71 ± 0.03a	149.2 ± 8.0b	–
	T1	6.16 ± 0.31a	8.04 ± 0.50a	10.6 ± 0.7b	0.72 ± 0.05a	36.6 ± 1.5c	0.68 ± 0.11a	150.7 ± 41.7b	–
	T2	6.15 ± 0.27a	9.93 ± 3.21a	14.9 ± 2.7a	0.82 ± 0.23a	45.9 ± 3.7b	0.66 ± 0.29a	179.8 ± 41.7ab	–
	T3	6.24 ± 0.37a	9.54 ± 2.21a	11.7 ± 2.0ab	0.75 ± 0.07a	55.3 ± 4.0a	0.79 ± 0.08a	216.6 ± 28.1a	–

CK, T1, T2, and T3 represent compost application at rates of 0, 15, 30, and 45 t ha^−1^, respectively. 0–20, 20–40, and 40–60 cm are the depths of the soil samples that were taken in the profile. Different lowercase letters in the same column indicate significant differences (*p* < 0.05) between treatments in each layer.

### Heavy metal accumulation and availability in soil

**Figure 1:**
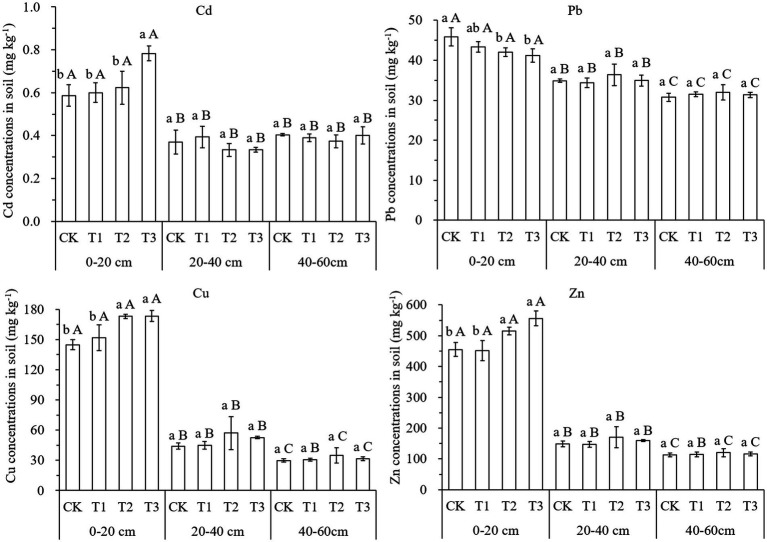
Effects of compost application on heavy metal concentrations in soil (mean ± S.D., *n* = 3). CK, T1, T2, and T3 mean compost application at rates of 0, 15, 30, and 45 t ha^−1^, respectively. 0–20, 20–40, and 40–60 cm are the depths of soil samples that were taken in the profile. The different lowercase letters indicate significant differences (*p* < 0.05) between treatments in each layer, while different capital letters indicate significant differences (*p* < 0.05) between soil layers in each treatment.

The application of compost significantly affected the accumulation of heavy metals in the topsoil, with the heavy metal concentrations varying significantly between the different soil layers (Figure 1). In the top layer of soil, the Cd concentration was increased by 32% only for the T3 treatment, Cu concentrations were increased by 20% for both the T2 and T3 treatments, Zn concentrations were increased by 13 and 22%, respectively, for the T2 and T3 treatments, while compared to CK, Pb concentrations in T2 and T3 were decreased by 8 and 10%, respectively. There were no significant changes in heavy metals between treatments in both the subsurface and the deep soil because of the compost application. The contents of Cd, Pb, Cu, and Zn in the topsoil were much higher than those in the subsurface and deep soil. Two-way ANOVA analysis further demonstrated that compost addition and soil depth had significant effects on heavy metal accumulation in the soil (Supplementary Table S1).

**Figure 2:**
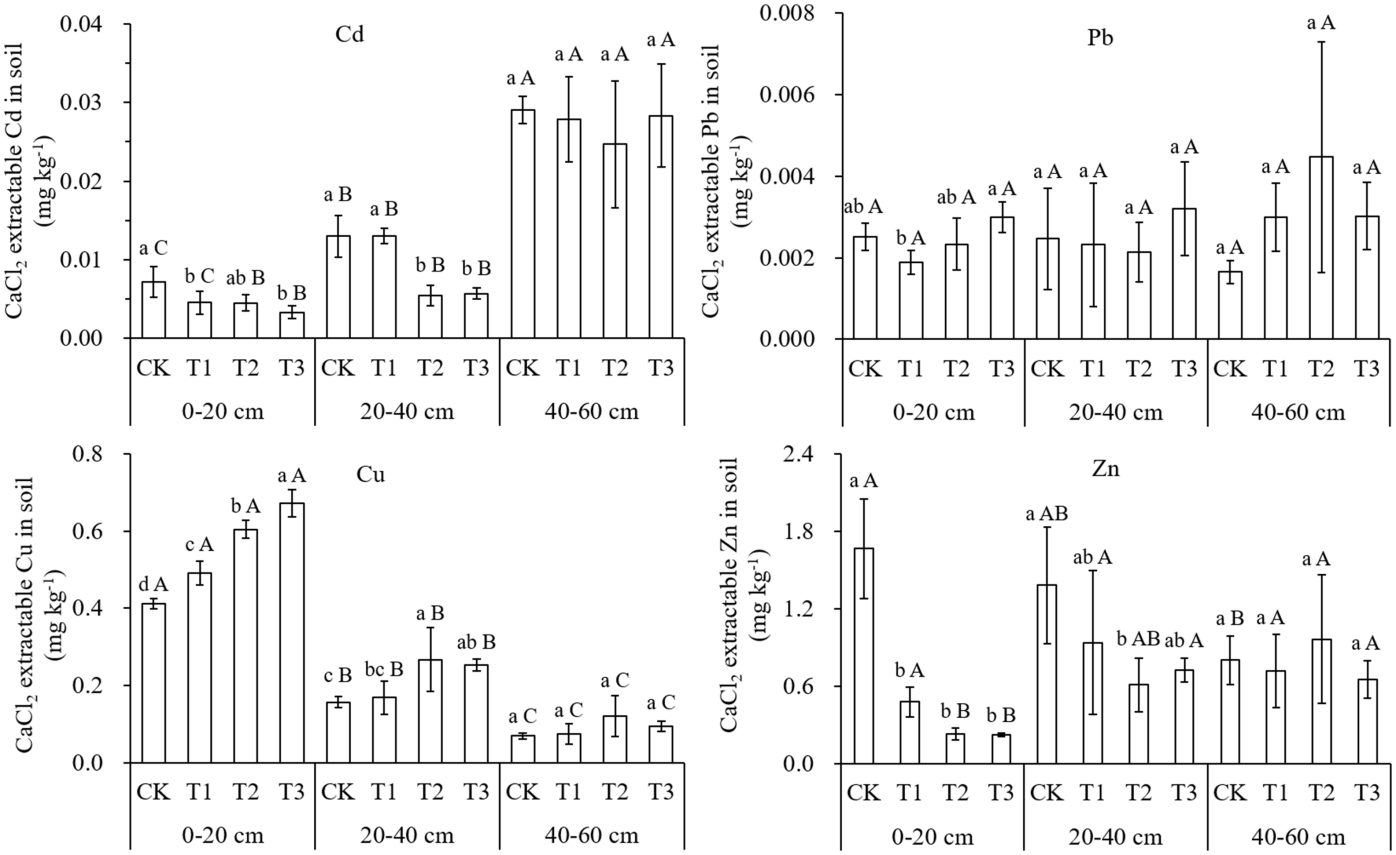
Effects of compost application on available concentration of heavy metals in soil (mean ± SD, *n* = 3). CK, T1, T2, and T3 show compost application at rates of 0, 15, 30, and 45 t ha^–1^, respectively. 0–20, 20–40, and 40–60 cm are the depths of soil samples that were taken in the profile. The different lowercase letters indicate significant differences (*p* < 0.05) between treatments in each layer, while different capital letters indicate significant differences (*p* < 0.05) between soil layers in each treatment.

Except for Pb levels, the available heavy metal contents varied significantly with compost application both in the surface and subsurface soil layers (Figure 2). The available Cd content was reduced by 38–54% and 56–58%, respectively, in the surface and subsoil because of the addition of compost. Contrary to the changes of total Cd concentration, the available Cd content in the topsoil was significantly lower than that in the subsurface and deep soil. The available Zn content decreased by 71–86 and 56%, respectively, in the surface and subsoil which were treated with manure compost. The available Zn content in the topsoil was twice that in the deep soil for the CK, while they were 0.24–0.35 folds in manure treatments. By comparison, available Cu content was increased by 19–63% and 61–70%, respectively, in the surface and subsoil because of the addition of compost, meanwhile, available Cu concentrations in the topsoil were much higher than those in the subsurface and deep soil (Figure 2). Two-way ANOVA analysis also indicated that the addition of compost significantly affected the available Cd, Cu, and Zn content in the soil, while there were significant differences between the layers for available Cd and Cu concentrations (Supplementary Table S2). There were no significant changes in available Pb content amongst the compost treatments or soil layers.

### Heavy metal concentration in asparagus lettuce

The application of compost has various effects on the heavy metal concentration in the various components of the lettuce plants (Table 3). Cd concentrations were reduced by 41–50%, 28–39%, and 31–39%, respectively in the shoots, leaves, and roots. Zn concentrations in the shoots, leaves, and roots of the plants were reduced by 14, 38–39%, and 45–67%, respectively, in compost treatments, compared to CK. Cu concentrations were increased by 20–39% in the lettuce shoots and decreased by 16–27% in the roots in compost treatments. There were no significant effects of compost application on the concentrations of Pb in any component of the plants. Compost application significantly increased the biomass of the lettuce plants, especially when compost was applied at high rates (T2 and T3). The dry weight of the shoots, leaves, and roots increased by 30–49%, 53–54%, and 45–59%, respectively, because of the application of various amounts of compost.

**Table 3:** Effects of compost addition on plant heavy metal concentrations and biomass.

Tissue	Treatment	Cd mg kg^–1^	Pb mg kg^–1^	Cu mg kg^–1^	Zn mg kg^–1^	Biomass (DW^*^, g plant^–1^)
Stems	CK	0.43 ± 0.04a	0.10 ± 0.04a	6.80 ± 0.96b	47.6 ± 3.4a	2.48 ± 0.12c
	T1	0.26 ± 0.04b	0.18 ± 0.02a	8.17 ± 0.49ab	40.9 ± 1.4b	2.71 ± 0.12bc
	T2	0.24 ± 0.01b	0.17 ± 0.05a	9.19 ± 0.68a	42.3 ± 1.0ab	3.68 ± 0.46a
	T3	0.22 ± 0.03b	0.16 ± 0.05a	9.45 ± 1.07a	40.8 ± 4.4b	3.22 ± 0.34ab
Leaves	CK	0.38 ± 0.02a	1.60 ± 0.10a	8.95 ± 0.90a	62.5 ± 18.4a	2.80 ± 0.23b
	T1	0.27 ± 0.08b	1.68 ± 0.31a	8.08 ± 1.16a	38.6 ± 3.0b	3.27 ± 0.28b
	T2	0.24 ± 0.04b	1.86 ± 0.42a	8.91 ± 1.93a	45.5 ± 5.5ab	4.28 ± 0.42a
	T3	0.23 ± 0.02b	1.53 ± 0.23a	7.71 ± 0.91a	37.9 ± 4.2b	4.32 ± 0.56a
Roots	CK	0.43 ± 0.05a	1.28 ± 0.31ab	24.9 ± 2.5a	149.0 ± 27.2a	0.44 ± 0.06b
	T1	0.30 ± 0.06b	1.65 ± 0.28a	20.8 ± 1.9b	81.7 ± 14.0b	0.68 ± 0.07a
	T2	0.26 ± 0.05b	1.43 ± 0.32ab	19.3 ± 2.4b	52.5 ± 8.3bc	0.64 ± 0.03a
	T3	0.26 ± 0.09b	1.03 ± 0.33b	18.2 ± 1.1b	49.2 ± 12.8c	0.70 ± 0.02a

* , DW means dry weight of the biomass.

CK, T1, T2, and T3 represent compost application at rates of 0, 15, 30, and 45 t ha^−1^, respectively. Different lowercase letters in the same column indicate significant differences (*p* < 0.05) between treatments in each component of the asparagus lettuce plant.

### Heavy metal translocation, bioconcentration and uptake in asparagus lettuce

**Figure 3:**
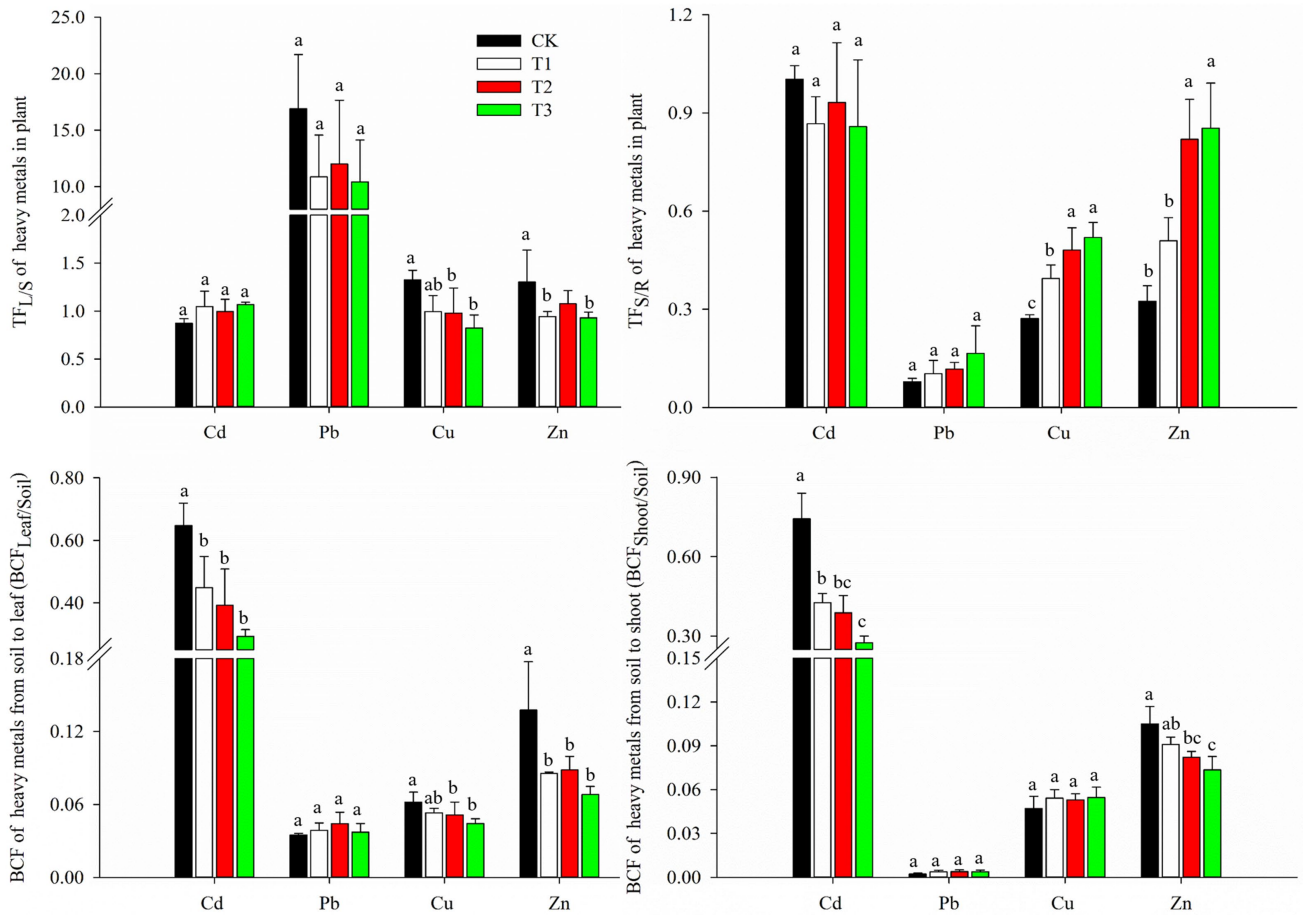
Effects of compost application on translocation factor and bioconcentration factor of heavy metals (mean ± SD, *n* = 3). TF_L/S_ is the translocation factor of heavy metals from shoot to leaf, TF_S/R_ is the translocation factor of heavy metals from root to shoot. BCF stands for the bioconcentration factor of heavy metals from soil to plant. CK, T1, T2, and T3 are compost application at rates of 0, 15, 30, and 45 t ha^−1^, respectively. The different lowercase letters indicate significant differences (*p* < 0.05) between treatments.

The translocation and bioconcentration factors of the heavy metals are shown in Figure 3. The translocation factors vary with different heavy metals and depending on which plant organ. The TF_L/S_ for Pb was much higher than that for Cu, Zn, and Cd, whereas the TF_S/R_ for Pb was much lower than that for Cd, Zn, and Cu. There were similar values of TF_L/S_ and TF_S/R_ for Cd, within the range of 0.87–1.07 and 0.86–1.00, respectively. The values of TF_L/S_ for Cu (0.82–1.32) and Zn (0.93–1.30) were both higher than their corresponding values for TF_S/R_ (0.27–0.52, and 0.32–0.85). There were no significant effects from the addition of compost on TF_L/S_ and TF_S/R_ for either Cd or Pb, compared to CK. However, compost treatments effectively reduced the TF_L/S_ for Cu and Zn by 26–38% and 27–28%, respectively, and increased the TF_S/R_ for Cu and Zn by 46–92% and 156–167%, respectively, compared to the control.

The order of BCF_Leaf/Soil_ and BCF_Shoot/Soil_ for heavy metals was as follows: Cd > Zn > Cu > Pb. The values of BCF_Leaf/Soil_ and BCF_Shoot/Soil_ for Cd were similar, as were those for Cu and Zn. The BCF_Leaf/Soil_ for Pb was approximately 10 times higher than that of BCF_Shoot/Soil_. Compost application effectively reduced the BCF_Leaf/Soil_ and BCF_Shoot/Soil_ of Cd by 42–63% and 31–55%, respectively. There were no differences in BCF_Leaf/Soil_ of Pb and Cu between treatments due to compost addition, as there were for the BCF_Shoot/Soil_ of Pb and Cu. In addition, compost treatments generally decreased the BCF_Shoot/Soil_ of Zn (18–26%), the BCF_Leaf/Soil_ of Cu (17–28%), and Zn (37–51%), compared to CK.

**Figure 4:**
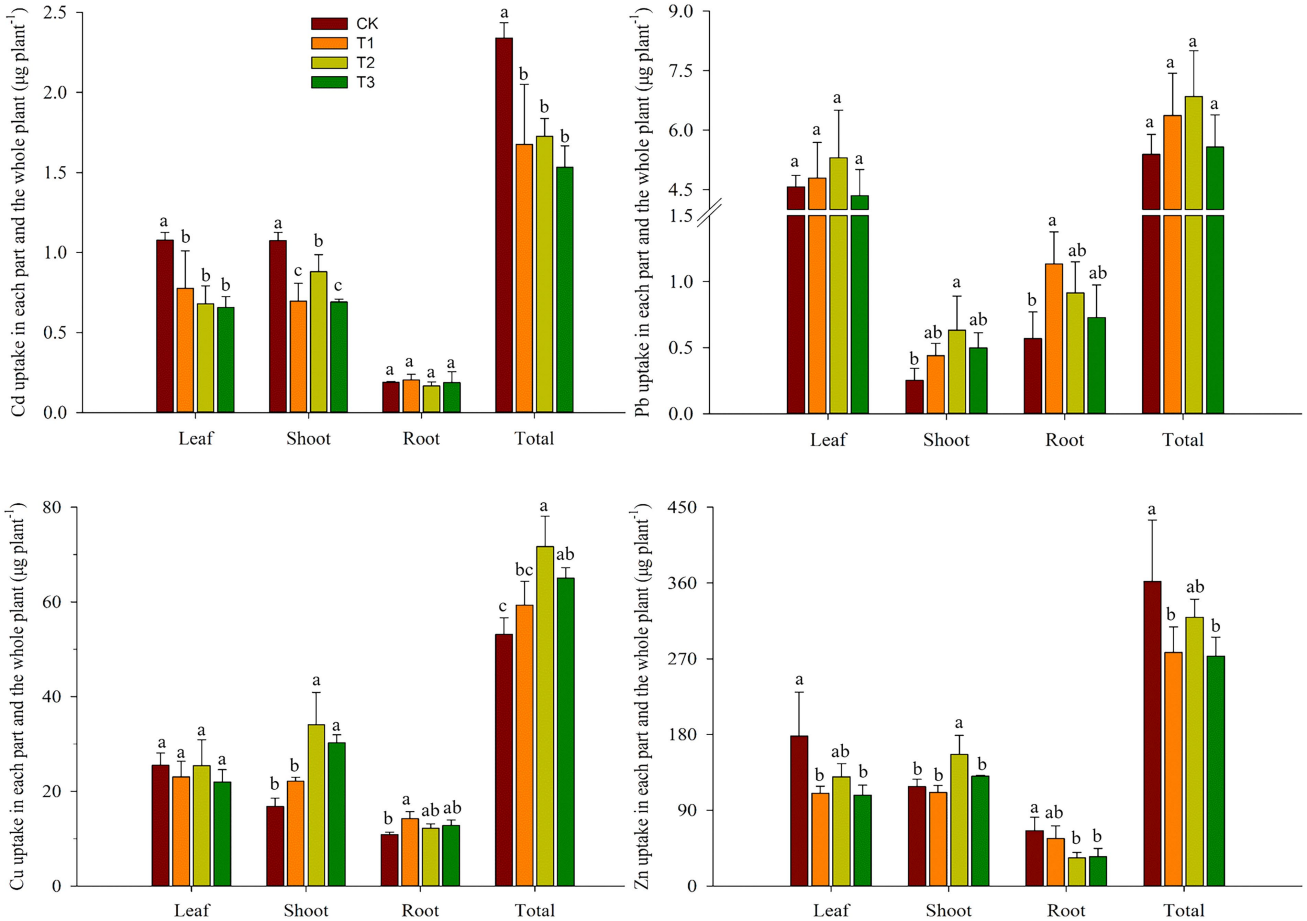
Effects of compost application on heavy metal uptake in each component as well as the entire plant (mean ± SD, *n* = 3). CK, T1, T2, and T3 are compost application at rates of 0, 15, 30, and 45 t ha^−1^, respectively. The different lowercase letters indicate significant differences (*p* < 0.05) between treatments.

Figure 4 shows the total uptake of heavy metals in each component of asparagus lettuce as well as the whole plant. The order of the total uptake of heavy metals in the entire plant was as follows: Zn > Cu > Pb > Cd. Cd uptake in leaves, shoots, and roots which accounts for 39–46%, 42–51%, and 8–12% of the whole plant, respectively. For Pb, the levels were 75–85%, 5–9%, and 11–18% in the leaves, shoots, and roots, respectively. Approximately 34–48%, 32–47%, and 17–24% of Cu, and 40–49%, 33–49%, and 10–20% of Zn accumulated in the leaves, shoots, and roots, respectively. Treatment with compost significantly reduced the uptake of Cd in leaves, shoots, and the whole plant by 28–39%, 18–36%, and 26–35%, respectively. There were no significant changes in the total Pb uptake in the whole plant. As a result of adding compost, the total Zn uptake was reduced by 38–39%, 47–49%, and 23–25% in asparagus lettuce leaves, roots, and whole plants, respectively, while there was a 32% increase in the shoots in the T2 treatment. The total Cu uptake in the shoots, roots, and whole plants generally increased by up to 103, 31, and 35%, respectively.

## Discussion

The present study was performed in a slightly acid soil, continuous application of compost significantly increased the pH of soil, indicating compost addition is effective in against soil acidification. The increase of soil pH may result from the alkaline characteristic of the compost. Besides, the application of organic fertilizer provides sufficient base ions for the soil, which can cope with the acidification of soil. Dai et al. (2021) found that the application of chemical fertilizer for 10 years led to a decrease of soil pH by 0.84 units, while the pH of organic fertilizer treatment was increased by 0.18 units. Besides, the application of organic fertilizer reduced the soil exchangeable acid content, and increased the total amount of exchangeable base compared to chemical fertilizer (Dai et al., 2021). Xiao et al. (2021) also reported that in soil pH values were significantly reduced by continuous application of chemical fertilizers compared with unfertilized treatment in a 28-year field experiment, while application of manure alone or combined with chemical fertilizer increased soil pH.

Compost application induced heavy metal accumulation in soil depends on the application rate, frequency and duration, as well as the heavy metal content of compost. In this study, there were no significant changes of heavy metals in topsoil when compost was applied at 15 t ha^−1^, which is close to the level of local farmers. When compost was added at 30 and 45 t ha^−1^, it increased the accumulation of Cu, Zn, and Cd in topsoil. Zhao et al. (2014) reported that the application of cattle manure for 10 years at 20 and 40 t ha^−1^ led to a dramatic increase in Cd and Cr, a slight decrease in Zn, and no changes in Pb in the top soil, compared to non-fertilizer treatment and chemical fertilizer treatment. Wang et al. (2020) found that the continuous addition of pig manure for 16 years at 1.6 t ha^−1^ each year resulted in the increased concentrations of Cu, Zn, and Cd, and reduced Pb in the soil compared with chemical fertilizer treatment, which is in accordance with this study. Our previous study investigated the heavy metal contents of 99 commercial organic fertilizers and found that the average concentrations of Cd, Pb, Cu, and Zn were 0.63, 17.3, 213.8, 660.5, mg kg^−1^, respectively (Ye et al., 2020), which was higher than the heavy metal contents of the compost used in this study. Wang et al. (2020) also found that Cd concentration in peanut kernels was significantly higher in soil treated with manure than in that treated with chemical fertilizer, which could be a result of the high content of Cd in the manure (7.91 mg kg^−1^). Soil texture is another factor that contributes to the accumulation and movement of heavy metals in soil. Scokart et al. (1983) found that loamy soils have a greater capacity for heavy metal accumulation than sandy soils. This study demonstrated that 15 t ha^−1^ is a rational application rate of compost as higher rates increase the risk of Cd, Cu, and Zn accumulation in topsoil.

The results show that heavy metals are more likely to accumulate in the topsoil throughout the entire soil profile, especially in the case of the long-term application of large amounts of compost (Figure 1). Two-way ANOVA analysis also showed that soil depth and the addition of compost had a significant effect on the concentration of each heavy metal in the soil (Supplementary Table S1). The migration coefficient of each heavy metal was greater than 1.0, further indicating that heavy metals accumulated more easily in the topsoil (Supplementary Figure S1). A previous study showed that the movement of heavy metals is affected by soil properties, including organic matter content, texture, and pH (Scokart et al., 1983). They found that the heavy metals would remain in the upper layers in loamy soils provided that the pH was higher than 6, with the mobility of Cd increasing when the pH was lower than 6 (with it being below 5 for Zn). The metals would then be associated with organic matter for migration (Scokart et al., 1983). The soil pH in this study became neutral after the addition of compost, which, when combined with the loamy texture, may be the reason there was no increase in heavy metals in the subsoil and deep soil. In general, Cu, Zn, and Cd accumulated more easily in the topsoil rather than Pb.

Although the concentrations of Cd, Cu, and Zn were increased in topsoil because of the addition of compost, the availability of Cd and Zn in the soil was significantly decreased, while the availability of Cu was increased compared to CK. The change in the availability of each heavy metal in the soil was generally consistent with the change in the heavy metal concentration in the leaves or shoots because of compost addition, which was further supported by correlation analysis (Supplementary Tables S3–S6). The decrease in soil Cd and Zn bioavailability may have resulted from the increase in soil pH, organic matter content, and CEC due to compost application. Soil pH is the most important factor which controls heavy metal availability by the determination of heavy metal speciation and solubility in the soil as a whole, particularly in the soil solution (Mühlbachová et al., 2005; Zhao et al., 2010; Chen et al., 2020). The increase in soil pH could increase the negative charge of the soil particles, which is beneficial for the absorption of heavy metal ions onto the soil (Naidu et al., 1998). In addition, the increased soil pH could promote the hydrolysis of Cd^2+^ and Pb^2+^ to form strongly bound hydroxyl species such as CdOH^+^ and PbOH^+^, which are more likely to be absorbed by soil (Lindsay, 1979; Bolan et al., 2003). In addition, the increase in soil pH may cause the formation of metal precipitates such as metal hydroxides, carbonates, and even phosphates, thereby decreasing the availability of heavy metals in soil (Liang et al., 2014; Huang et al., 2017).

The increased SOM in the compost treatments in this study might also contribute to the decreased availability of Cd and Zn in the soil and the subsequent plant uptake. SOM is rich in carboxyl and hydroxyl groups that can react with metal ions (Kwiatkowska-Malina, 2018). Lair et al. (2007) found that the sorption of Cu, Cd, and Zn on organic matter exceeded the mineral sorption by 6 to 13 times in soil, thus indicating the role of SOM in the retention of heavy metals. Conversely, it has been reported that heavy metal adsorption onto soil decreases with the decline in SOM (Hettiarachchi et al., 2003; Antoniadis et al., 2008). There was a significant negative correlation between SOM and Cd concentration in each plant part, as shown in Supplementary Figure S3. In addition, there were significant increases in soil CEC, which also contributed to the retention of heavy metals in the soil (Sungur et al., 2014). It is difficult to distinguish the contribution of CEC alone because the increases in soil pH and SOM also stimulated soil CEC (Supplementary Table S3).

In this study, the changes in Cu availability in soil and its concentration in plants were inconsistent with those of Cd. The increase in Cu bioavailability may be explained by the fact that Cu^2+^ readily reacts with dissolved organic matter (DOM) in the soil compared to Cd^2+^ (Temminghoff et al., 1997; Ginocchio et al., 2002), while the addition of compost into soil can increase the concentrations of soil DOM (Welikala et al., 2021). In addition, increased soil pH promotes the dissolution of SOM, further increasing the content of DOM in the soil (Oste et al., 2002). Ashworth and Alloway (2008) reported that the solubility of Cu, Ni, and Pb showed a strong positive relationship to the solubility of organic matter in the soil, particularly at a high pH. McBride and Blasiak (1979) found that the complex Cu^2+^ fraction in the soil solution increased dramatically with the pH value, and accounted for up to 99.9% of the total soluble Cu^2+^ at pH 8, indicating that most of the soluble Cu existed in complex forms. In this study, the addition of compost to acidic soil increased the soil pH to a neutral range, which may have induced the complexation of Cu^2+^ with DOM, thereby promoting the mobility and availability of Cu. A previous study showed that DOM-compounds are generally more significant for Cu and Pb than for Cd, Zn, and Ni (Weng et al., 2002). In addition, the structural properties of DOM are another important factor that determines the mobilization of heavy metals from soil (Welikala et al., 2021).

The bioconcentration factor reflects the ability of plants to accumulate heavy metals from the soil. Root to shoot translocation (TF_S/R_) is a key process determining heavy metal accumulation in the aboveground portions of plants after root uptake (Uraguchi et al., 2009; Yu and Zhou, 2009). Both the BCF_leaf/soil_ and BCF_Shoot/Soil_ of Cd were much higher than those of other elements, indicating that Cd is more likely to accumulate in Asparagus lettuce. In addition, the TF_S/R_ and TF_L/S_ of Cd were approximately 1.0, demonstrating the strong ability of Asparagus lettuce plants to transport Cd from their roots to the shoots and leaves. According to the results of the BCF and TF, compost application reduced the uptake of Cd from soil to Asparagus lettuce but did not affect the translocation of Cd from the roots to the aboveground portion. However, with Cu and Zn, compost reduced their concentration ability, increased their translocation from roots to shoots, and decreased their translocation from shoots to leaves, suggesting that the addition of compost facilitates the accumulation of Cu and Zn in lettuce shoots. Pb has a lesser ability to enter the roots and further translocate to the shoots, while having a strong ability to translocate from the shoots to the leaves. The application of compost effectively increased the biomass of each section of the lettuce due to improved soil fertility. The total uptake quantities of Cd in the leaves, shoots, roots, and entire plant was significantly decreased in the compost treatments compared to CK. Therefore, there was a possible dilution of Cd concentrations in the Asparagus lettuces, as there were significant increases in biomass. As for Cu, the total uptake amount in the shoots and the entire plant significantly increased because of the addition of compost.

## Conclusion

Application of compost at 15–45 t ha^−1^ for 12 consecutive growing seasons decreased soil acidity and improved soil fertility. The accumulation of Cd, Cu, and Zn in the topsoil (0–20 cm) were increased by up to 32, 20, and 22%, respectively, while Pb concentrations were reduced by 8–10%. However, available Cd and Zn concentrations were reduced by 38–54% and 71–86%, respectively, and available Cu was increased by 19–63% in the topsoil in the compost treatment. There was no significant effect of compost on the available Pb content. Compost application effectively increased the biomass of asparagus lettuce by 30–59%. In compost treatments, Cd and Zn concentrations in the roots, shoots, and leaves of the lettuce were reduced by 28–50% and 14–67%, respectively, while Cu concentrations were increased by 20–39% in the shoots. There were no significant changes in the levels of Pb in any part of the plant compared to CK. This study demonstrated that 15 t ha^−1^ is a rational application rate of compost as higher rates increase the risk of Cd, Cu, and Zn accumulation in topsoil.

## Data availability statement

The original contributions presented in the study are included in the article/Supplementary material, further inquiries can be directed to the corresponding author.

## Author contributions

DC: conceptualization, data curation, and writing—reviewing and editing. XY: conceptualization, supervision, and reviewing and editing. YJ and SS: field experiment and sampling. WX: sampling, data curation, and software. QZ: visualization and software. SZ: sample preparation. NG: detection of heavy metals in plant samples. MH: determination of heavy metals in soil samples. JH: testing of physico-chemical properties of soil samples. All authors contributed to the article and approved the submitted version.

## Funding

This work was supported by the National Key Research and Development Program of China (grant number 2018YFF0213501-2), Science and Technology Cooperation Project of Agriculture and Rural Department of Zhejiang Province (grant number 2020SNLF004), and Major Agriculture Science and Technology Projects of Zhejiang Province, China (grant number 2015C02042).

## Conflict of interest

The authors declare that the research was conducted in the absence of any commercial or financial relationships that could be construed as a potential conflict of interest.

## Publisher’s note

All claims expressed in this article are solely those of the authors and do not necessarily represent those of their affiliated organizations, or those of the publisher, the editors and the reviewers. Any product that may be evaluated in this article, or claim that may be made by its manufacturer, is not guaranteed or endorsed by the publisher.

## Supplementary material

The Supplementary Material for this article can be found online at: https://www.frontiersin.org/articles/10.3389/fpls. 2022.972789/full#supplementary-material
