# Supplementary material for: Continuously applying compost for three years alleviated soil acidity and heavy metal bioavailability in a soil-asparagus lettuce system
Source: Front Plant Sci. 2022 Aug 3;13:972789. doi: 10.3389/fpls.2022.972789 (PMC9390081; doi:10.3389/fpls.2022.972789)
Supplement: Supplementary file 1 [file Data_Sheet_1.DOCX]

**Continuously applying compost for three years alleviated soil acidity and heavy metal bioavailability in a soil-asparagus lettuce system**

De Chen^a^, Xuezhu Ye^a,^ *, Yugen Jiang^b^, Wendan Xiao^a^, Qi Zhang^a^, Shouping Zhao^a^, Sainan Shao^b^, Na Gao^a^, Miaojie Huang^a^, Jing Hu^a^

^a^ State Key Laboratory for Managing Biotic and Chemical Threats to the Quality and Safety of Agro-products; Key Laboratory of Information Traceability for Agricultural Products, Ministry of Agriculture and Rural Affairs of China; Institute of Agro-product Safety and Nutrition, Zhejiang Academy of Agricultural Sciences, Hangzhou 310021, Zhejiang, China

^b^ Agricultural Technology Extension Center of Fuyang District*,* Hangzhou 311400, Zhejiang, China

*** Corresponding Author:** Xuezhu Ye, Professor

Address: Institute of Agro-product Safety and Nutrition, Zhejiang Academy of Agricultural Sciences, No. 298 Desheng Road, Hangzhou 310021, China

Tel: +86-0571-86415206; Fax: +86-0571-86450124

E-mail: rosecomepaper@163.com

**Table S1.** Two-way ANOVA of the effects of compost and soil depth on heavy metal accumulation

| Dependent variable | Source | type III Sum of squares | df | Mean square | F | Sig. |
| --- | --- | --- | --- | --- | --- | --- |
| Total Cd content in  soil | Corrected model | .691^a^ | 11 | .063 | 35.443 | .000 |
|  | intercept | 7.812 | 1 | 7.812 | 4408.039 | .000 |
|  | depth * manure | .064 | 6 | .011 | 6.025 | .001 |
|  | depth | .607 | 2 | .303 | 171.118 | .000 |
|  | manure | .020 | 3 | .007 | 3.830 | .023 |
|  | Error | .043 | 24 | .002 |  |  |
|  | Total | 8.545 | 36 |  |  |  |
|  | Corrected Total | .733 | 35 |  |  |  |
| Total Pb content in  soil | Corrected model | 906.95^b^ | 11 | 82.45 | 36.15 | .000 |
|  | intercept | 48077.87 | 1 | 48077.87 | 21079.08 | .000 |
|  | depth * manure | 38.32 | 6 | 6.39 | 2.80 | .033 |
|  | depth | 859.52 | 2 | 429.76 | 188.42 | .000 |
|  | manure | 9.10 | 3 | 3.03 | 1.33 | .288 |
|  | Error | 54. | 24 | 2.28 |  |  |
|  | Total | 49039.56 | 36 |  |  |  |
|  | Corrected Total | 961.69 | 35 |  |  |  |
| Total Cu content in  soil | Corrected model | 119560.97^c^ | 11 | 10869.18 | 227.05 | .000 |
|  | intercept | 234707.95 | 1 | 234707.96 | 4902.94 | .000 |
|  | depth * manure | 813.76 | 6 | 135.63 | 2.83 | .031 |
|  | depth | 117226.62 | 2 | 58613.31 | 1224.40 | .000 |
|  | manure | 1520.59 | 3 | 506.86 | 10.59 | .000 |
|  | Error | 1148.90 | 24 | 47.87 |  |  |
|  | Total | 355417.82 | 36 |  |  |  |
|  | Corrected Total | 120709.87 | 35 |  |  |  |
| Total Zn content in  soil | Corrected model | 1061193.53^d^ | 11 | 96472.14 | 285.02 | .000 |
|  | intercept | 2351111.11 | 1 | 2351111.11 | 6946.20 | .000 |
|  | depth * manure | 12874.25 | 6 | 2145.71 | 6.34 | .000 |
|  | depth | 1037154.84 | 2 | 518577.42 | 1532.10 | .000 |
|  | manure | 11164.44 | 3 | 3721.48 | 11.00 | .000 |
|  | Error | 8123.38 | 24 | 338.47 |  |  |
|  | Total | 3420428.02 | 36 |  |  |  |
|  | Corrected Total | 1069316.91 | 35 |  |  |  |

a. R^2^ = .942(Adjusted R^2^ = .915); b. R^2^ = .943(Adjusted R^2^ = .917); c. R^2^ = .990(Adjusted R^2^ = .986); d. R^2^ = .943(Adjusted R^2^ = .917)

**Table S2.** Two-way ANOVA of the effects of compost and soil depth on heavy metal availability

| Dependent variable | Source | type III Sum of squares | df | Mean square | F | Sig. |
| --- | --- | --- | --- | --- | --- | --- |
| Available Cd content in soil | Corrected model | .004^a^ | 11 | .000 | 25.261 | .000 |
|  | intercept | .007 | 1 | .007 | 533.91 | .000 |
|  | depth * manure | 8.16E-005 | 6 | 1.360E-005 | 1.037 | .426 |
|  | depth | .003 | 2 | .002 | 130.77 | .000 |
|  | manure | .000 | 3 | 4.419E-005 | 3.37 | .035 |
|  | Error | .000 | 24 | 1.311E-005 |  |  |
|  | Total | .011 | 36 |  |  |  |
|  | Corrected Total | .004 | 35 |  |  |  |
| Available Pb content in soil | Corrected model | 1.65E-005^b^ | 11 | 1.503E-006 | .983 | .487 |
|  | intercept | .000 | 1 | .000 | 174.43 | .000 |
|  | depth * manure | 1.10E-005 | 6 | 1.842E-006 | 1.21 | .337 |
|  | depth | 1.40E-006 | 2 | 7.015E-007 | .46 | .637 |
|  | manure | 4.07E-006 | 3 | 1.357E-006 | .89 | .461 |
|  | Error | 3.67E-005 | 24 | 1.528E-006 |  |  |
|  | Total | .000 | 36 |  |  |  |
|  | Corrected Total | 5.32E-005 | 35 |  |  |  |
| Available Cu content in soil | Corrected model | 1.49^c^ | 11 | .135 | 102.50 | .000 |
|  | intercept | 2.86 | 1 | 2.865 | 2171.90 | .000 |
|  | depth * manure | .047 | 6 | .008 | 5.96 | .001 |
|  | depth | 1.33 | 2 | .666 | 505.14 | .000 |
|  | manure | .11 | 3 | .036 | 27.16 | .000 |
|  | Error | .03 | 24 | .001 |  |  |
|  | Total | 4.38 | 36 |  |  |  |
|  | Corrected Total | 1.52 | 35 |  |  |  |
| Available Zn content in soil | Corrected model | 5.88^d^ | 11 | .534 | 5.77 | .000 |
|  | intercept | 22.14 | 1 | 22.14 | 239.10 | .000 |
|  | depth * manure | 2.31 | 6 | .38 | 4.16 | .005 |
|  | depth | .42 | 2 | .21 | 2.26 | .126 |
|  | manure | 3.15 | 3 | 1.05 | 11.33 | .000 |
|  | Error | 2.22 | 24 | .09 |  |  |
|  | Total | 30.24 | 36 |  |  |  |
|  | Corrected Total | 8.10 | 35 |  |  |  |

a. R^2^ = .920(Adjusted R^2^ = .884); b. R^2^ = .311(Adjusted R^2^ =-.005); c. R^2^ = .979(Adjusted R^2^ = .970); d. R^2^ = .726(Adjusted R^2^ = .600)


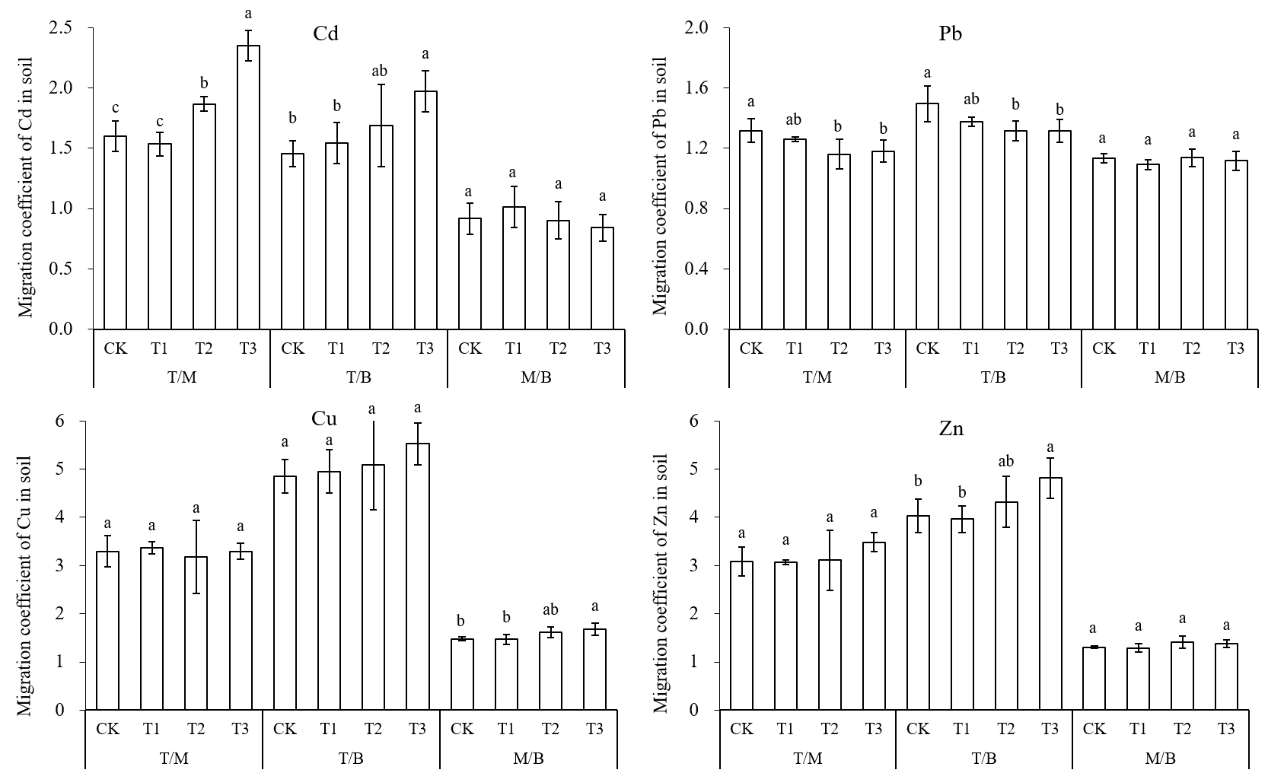


Fig. S1

| **Table S3.** Pearson correlation of Cd bioavailability and soil properties | | | | | | | | | |
| --- | --- | --- | --- | --- | --- | --- | --- | --- | --- |
|  | | AvaCd | pH | SOM | CEC | Shoot Cd | Leaf Cd | Root Cd | |
| AvaCd | r | 1 |  |  |  |  |  |  |  |
|  | Sig. |  |  |  |  |  |  |  |  |
| pH | r | -.437 | 1 |  |  |  |  |  |  |
|  | Sig. | .155 |  |  |  |  |  |  |  |
| SOM | r | -.693^*^ | .633^*^ | 1 |  |  |  |  |  |
|  | Sig. | .012 | .027 |  |  |  |  |  |  |
| CEC | r | -.619^*^ | .645^*^ | .899^**^ | 1 |  |  |  |  |
|  | Sig. | .032 | .024 | .000 |  |  |  |  |  |
| ShootCd | r | .779^**^ | -.763^**^ | -.829^**^ | -.796^**^ | 1 |  |  |  |
|  | Sig. | .003 | .004 | .001 | .002 |  |  |  |  |
| LeafCd | r | .654^*^ | -.652^*^ | -.762^**^ | -.773^**^ | .928^**^ | 1 |  |  |
|  | Sig. | .021 | .022 | .004 | .003 | .000 |  |  |  |
| RootCd | r | .566 | -.677^*^ | -.666^*^ | -.790^**^ | .863^**^ | .860^**^ | 1 |  |
|  | Sig. | .055 | .016 | .018 | .002 | .000 | .000 |  |  |
| *. Significant correlation at 0.05 level (bilateral); | | | | | | | | | |
| **. Significant correlation at 0.01 level (bilateral). | | | | | | | | | |

| **Table S4.** Pearson correlation of Pb bioavailability and soil properties | | | | | | | | | |
| --- | --- | --- | --- | --- | --- | --- | --- | --- | --- |
|  | | Ava Pb | pH | SOM | CEC | Shoot Pb | Leaf Pb | Root Pb | |
| Ava Pb | r | 1 |  |  |  |  |  |  |  |
|  | Sig. |  |  |  |  |  |  |  |  |
| pH | r | -.096 | 1 |  |  |  |  |  |  |
|  | Sig. | .766 |  |  |  |  |  |  |  |
| SOM | r | .363 | .633^*^ | 1 |  |  |  |  |  |
|  | Sig. | .247 | .027 |  |  |  |  |  |  |
| CEC | r | .220 | .645^*^ | .899^**^ | 1 |  |  |  |  |
|  | Sig. | .491 | .024 | .000 |  |  |  |  |  |
| Shoot Pb | r | .203 | .708^**^ | .428 | .319 | 1 |  |  |  |
|  | Sig. | .527 | .010 | .165 | .313 |  |  |  |  |
| Leaf Pb | r | -.338 | .165 | .061 | .227 | -.196 | 1 |  |  |
|  | Sig. | .283 | .608 | .850 | .477 | .542 |  |  |  |
| Root Pb | r | -.295 | .055 | -.257 | -.239 | .226 | .489 | 1 |  |
|  | Sig. | .352 | .865 | .419 | .454 | .481 | .107 |  |  |
| *. Significant correlation at 0.05 level (bilateral); | | | | | | | | | |
| **. Significant correlation at 0.01 level (bilateral). | | | | | | | | | |

| **Table S5.** Pearson correlation of Cu bioavailability and soil properties | | | | | | | | | |
| --- | --- | --- | --- | --- | --- | --- | --- | --- | --- |
|  | | Ava Cu | pH | SOM | CEC | Shoot Cu | Leaf Cu | Root Cu | |
| Ava Cu | r | 1 |  |  |  |  |  |  |  |
|  | Sig. |  |  |  |  |  |  |  |  |
| pH | r | .615^*^ | 1 |  |  |  |  |  |  |
|  | Sig. | .033 |  |  |  |  |  |  |  |
| SOM | r | .982^**^ | .633^*^ | 1 |  |  |  |  |  |
|  | Sig. | .000 | .027 |  |  |  |  |  |  |
| CEC | r | .871^**^ | .645^*^ | .899^**^ | 1 |  |  |  |  |
|  | Sig. | .000 | .024 | .000 |  |  |  |  |  |
| Shoot Cu | r | .720^**^ | .783^**^ | .771^**^ | .668^*^ | 1 |  |  |  |
|  | Sig. | .008 | .003 | .003 | .018 |  |  |  |  |
| Leaf Cu | r | -.255 | -.155 | -.195 | -.014 | -.217 | 1 |  |  |
|  | Sig. | .424 | .630 | .543 | .965 | .498 |  |  |  |
| Root Cu | r | -.750^**^ | -.650^*^ | -.792^**^ | -.852^**^ | -.550 | .272 | 1 |  |
|  | Sig. | .005 | .022 | .002 | .000 | .064 | .393 |  |  |
| *. Significant correlation at 0.05 level (bilateral); | | | | | | | | | |
| **. Significant correlation at 0.01 level (bilateral) | | | | | | | | | |

| **Table S6.** Pearson correlation of Zn bioavailability and soil properties | | | | | | | | | |
| --- | --- | --- | --- | --- | --- | --- | --- | --- | --- |
|  | | Ava Zn | pH | SOM | CEC | Shoot Zn | Leaf Zn | Root Zn | |
| Ava Zn | r | 1 |  |  |  |  |  |  |  |
|  | Sig. |  |  |  |  |  |  |  |  |
| pH | r | -.856^**^ | 1 |  |  |  |  |  |  |
|  | Sig. | .000 |  |  |  |  |  |  |  |
| SOM | r | -.820^**^ | .633^*^ | 1 |  |  |  |  |  |
|  | Sig. | .001 | .027 |  |  |  |  |  |  |
| CEC | r | -.759^**^ | .645^*^ | .899^**^ | 1 |  |  |  |  |
|  | Sig. | .004 | .024 | .000 |  |  |  |  |  |
| Shoot Zn | r | .772^**^ | -.577^*^ | -.565 | -.528 | 1 |  |  |  |
|  | Sig. | .003 | .049 | .056 | .078 |  |  |  |  |
| Leaf Zn | r | .840^**^ | -.564 | -.546 | -.459 | .799^**^ | 1 |  |  |
|  | Sig. | .001 | .056 | .066 | .133 | .002 |  |  |  |
| Root Zn | r | .973^**^ | -.843^**^ | -.850^**^ | -.801^**^ | .774^**^ | .828^**^ | 1 |  |
|  | Sig. | .000 | .001 | .000 | .002 | .003 | .001 |  |  |
| *. Significant correlation at 0.05 level (bilateral); | | | | | | | | | |
| **. Significant correlation at 0.01 level (bilateral) | | | | | | | | | |
